# Promyelocytic Blast Crisis of Chronic Myeloid Leukemia in a Patient Undergoing Therapy with a Tyrosine Kinase Inhibitor

**DOI:** 10.7759/cureus.7217

**Published:** 2020-03-09

**Authors:** Meghana Parsi, Tulin Budak-Alpdogan

**Affiliations:** 1 Internal Medicine, Crozer-Chester Medical Center, Upland, USA; 2 Hematology/Oncology, Cooper University Hospital, Camden, USA

**Keywords:** chronic myeloid leukemia, cml, acute promyelocytic leukemia, apl, blast crisis, tyrosine kinase inhibitor, tki

## Abstract

A 58-year-old male with the chronic phase of chronic myeloid leukemia (CML), treated with a tyrosine kinase inhibitor (TKI), bosutinib, since the past two years, presented with bright red bleeding per rectum and disseminated intravascular coagulation. A bone marrow biopsy reverse transcription-polymerase chain reaction revealed a promyelocytic blast crisis, with leukemic cells displaying both *BCR/ABL* and *PML/RAR*α chimeric genes. Cytogenetic studies revealed translocations of both t(15;17) and t(9;22). With the initiation of all-*trans* retinoic acid, arsenic trioxide and gemtuzumab, the patient achieved remission, with absent *PML/RAR*α by fluorescence in situ hybridization analysis. This case highlights the importance of long-term monitoring of patients with CML, especially those on TKIs, for the development of secondary leukemias in the future.

## Introduction

Chronic myeloid leukemia (CML) is an indolent neoplasm that can transform into acute leukemia, usually two to five years after diagnosis. Imatinib, dasatanib and bosutinib are all tyrosine kinase inhibitors (TKIs), known to produce remission in many patients. The promyelocytic blast crisis of CML is an extremely rare occurrence. In literature, there are only a limited number of such cases, dating as far back as 1986 [1,2]. A thorough literature review has revealed only two cases of promyelocytic blast crisis during treatment with a TKI. The first occurred in a 66-year-old woman with CML, nine months into treatment, and the other in a 50-year-old man, 12 months into treatment [1,3]. We present here a case of acute promyelocytic leukemia (APL) with associated disseminated intravascular coagulation (DIC) which developed two years into bosutinib treatment in a patient with chronic phase CML. 

## Case presentation

A 58-year-old male with a history of CML diagnosed in 2017, currently on the TKI bosutinib, presented with a several week history of fatigue, malaise, melena and bright red bleeding per rectum. Vital signs revealed a blood pressure of 130/78 mmHg, a heart rate tachycardiac at 102 beats per minute and a respiratory rate tachypneic at 22 cycles per minute. Pertinent physical exam findings included a well healing tracheostomy decannulation site as well as dry gangrenous right foot toes. Labs on day of admission are illustrated in Table 1. He also had evidence of coagulopathy with clinical and laboratory studies consistent with DIC.

**Table 1:** Significant labs

Parameter (normal range)	Labs on day of admission
Hemoglobin (14-18)	6.5 g/dL
White blood cell (WBC) (4.8-10.8)	10,000 cells/mm^3 ^(50% peripheral blasts)
Platelets (145-400x10^3^)	5,000
International normalized ratio (INR) (0.9-1.1)	1.75
Prothrombin time (PT) (11.8-14.7)	20.1 seconds
Partial thromboplastin time (PTT) (22-37)	26 seconds
Fibrinogen (200-400)	203 mg/dL
D-Dimer (<500)	56,713 ng/mL

**Figure 1:**
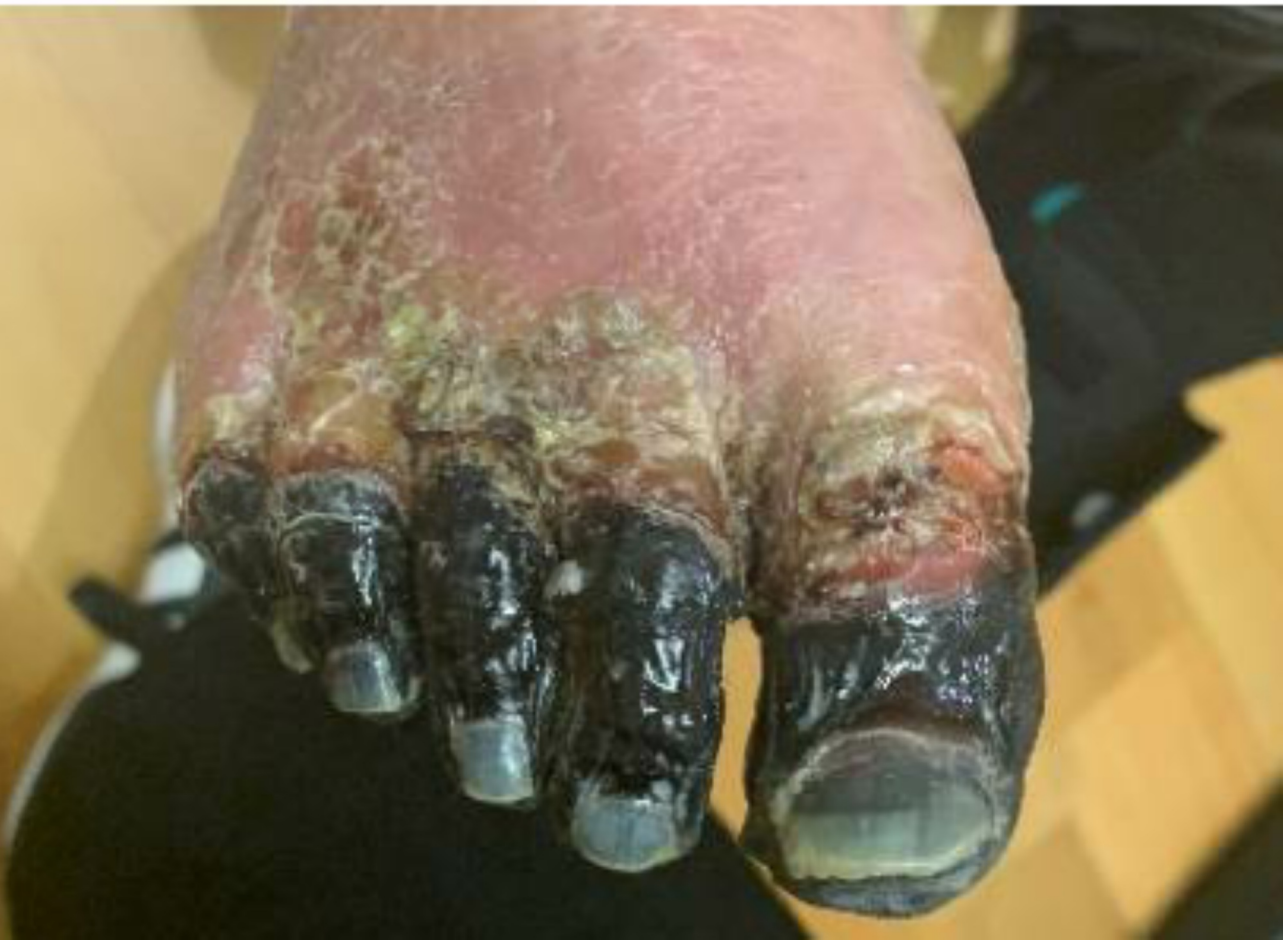
Necrosis of all five toes on the right foot

A bone marrow biopsy was hypercellular (90%) with 95% abnormal promyelocytes (myeloblasts). Flow cytometry analysis of the abnormal promyelocytes revealed immune positivity for CD4, CD13, CD33, CD38, CD64 and CD117 and immune negativity for MPO and HLA-DR expression. Karyotyping revealed 46,XY, t(9;22) (q34;q11.2) t(15;17)(q24;q21) del (17)(q23) in all 24 cells examined in the metaphase stage. Fluorescence in situ hybridization (FISH) analysis of the bone marrow aspirate demonstrated the typical PML/RARα and BCR-ABL1 chimeric gene translocations in 88% and 90% of the cells, respectively. FISH was negative for del 5q/5, 7q/7, 17p (TP53) or trisomy 8. Further molecular cytogenetics revealed mutations in the genes ASXL1, IKZF1 and WT1, signifying a poor prognosis. These findings were consistent with the acute promyelocytic blast crisis of CML. The patient was subsequently started on induction therapy with all-trans retinoic acid 45 mg/m^2^, arsenic trioxide 0.15 mg/kg IV daily, and gemtuzumab 6 mg/m^2^. The hospital stay was complicated by acute right-sided weakness secondary to acute intracranial hemorrhage in the left frontal, parietal and anterior cerebral artery territory. He was treated symptomatically as this bleed was believed to be secondary to DIC and his profound thrombocytopenia. He also developed a spontaneous left eye bullous subconjunctival hemorrhage along with bilateral ischemic toes, which eventually required amputation (Figure 1).

With an eventual resolution of his renal failure and improvement of his neurological function, he was discharged to a subacute rehabilitation facility. A repeat bone marrow biopsy, one month into treatment, showed no evidence of residual APL. 

## Discussion

CML is a myeloproliferative neoplasm of the hematopoietic stem cell characterized by dysregulated and uncontrolled proliferation of mature granulocytes. A reciprocal translocation between chromosomes 9 and 22, t(9;22)(q34;q11), gives rise to an abnormal fusion between BCR (on chromosome 22) and ABL1 (on chromosome 9) resulting in the BCR-ABL1 fusion gene. CML is characterized by the presence of the Philadelphia chromosome, which is present in >95% of cases. APL, on the other hand, is a separate and specific type of acute myeloid leukemia characterized by the presence of a fusion between PML (on chromosome 15q) and the RARα (on chromosome 17q), caused by t (15;17). Transformation from the chronic phase of CML to CML blast crisis develops over a range of nine months to nine years [4]. The time to development of promyelocytic blastic crisis was about two years in our patient. Unfortunately, the onset was characterized by coagulopathy with findings consistent with DIC. The exact mechanism behind blastic transformation is unclear, but several theories have been proposed. In patients treated with TKI, there is still a subset of patients (around 20%-25%) who do not achieve complete cytogenetic remission (CCyR), or absent (Ph+) positive cells in the marrow. Loss of response occurs in another 25% of patients who had initially responded to imatinib and achieved complete hematological response [5]. In these patients with TKI resistance, it is believed that uncontrolled activity if BCR/ABL in hematopoietic stem cells is responsible for disease progression [6]. Calabretta and Perrotti stated the presence of secondary genetic abnormalities that are responsible for the progression to a blast phase [4]. In a study conducted by Ruff et al., 75 cases of blast crisis were evaluated. They identified not only the presence of the Ph chromosome positive, but also new cytogenetic abnormalities in 52 patients. The most common anomalies included isochromosome of the long arm of chromosome 17 (i(17q), trisomies of chromosome 8, 19, 21, extra Ph chromosomes, among other deletions and mutations. A majority of the patients (54/75) had a myeloblastic transformation, while only three had a promyelocytic transformation [7]. It is also possible there was a small chimeric mRNA clone of PML/RARα at the time of initial diagnosis of CML. With the initiation of TKI, there may have been a shift in the clonal evolution, favoring the proliferation of leukemic cells harboring both chimeric genes. This was supported in a case by Chung et al., who presented a case of APL blastic transformation in a patient with CML nine months after treatment with imatinib. The bone marrow sample at the initial diagnosis of CML was tested by reverse transcription polymerase chain reaction, which revealed a chimeric PML/RARα mRNA value of 0.000321 [8]. With the elimination of the BCR-ABL1 containing leukemic cells, there was an opportunity for the proliferation of PML/RARα containing leukemic cells. A similar proposition was made by Oku et al., who also found the presence of PML/RARα several months before the onset of blast crisis [3]. O'Dwyer et al. proposed that prolonged treatment with a TKI led to the development of a clonal group of mutated progenitor cells, due to a suppressive effect on normal stem cells [9]. However, studies do not support TKIs to have a direct leukemogenic potential. Asciminib is a novel allosteric inhibitor of the myristoyl site on the BCR-ABL1 protein, being evaluated for the treatment of CML-CP in patients who have resistance/intolerance with more than two ATP-binding site TKIs. This halts BCR-ABL1 into an inactive conformation. This mechanism of action is different from that of the original TKIs. In 2017, the NCCN introduced a color-coded response chart that helped physicians to decide whether to switch to a new treatment or continue with current treatment. In the 2018 guidelines, patients with BCR-ABL1 levels >10% after six months of therapy with a TKI recommend switching to an alternative treatment [10].

## Conclusions

Serial measurements of BCR-ABL1 levels in patients and appropriate and timely treatment adjustments can curtail the development of accelerated disease or blast phase. Physicians must also be diligent and focus on additional abnormal clones at the initial diagnosis of CML, which may have the potential to advance into leukemia years later. The biological and clinical features of APL transformation in patients who have received TKIs are not fully explained or understood. We hope that this case report will help launch further research into this matter and to determine optimal salvage treatment in this very challenging setting.
